# Somatic Accumulation of GluA1-AMPA Receptors Leads to Selective Cognitive Impairments in Mice

**DOI:** 10.3389/fnmol.2018.00199

**Published:** 2018-06-25

**Authors:** David M. Bannerman, Thilo Borchardt, Vidar Jensen, Andrey Rozov, Nadia N. Haj-Yasein, Nail Burnashev, Daniel Zamanillo, Thorsten Bus, Isabel Grube, Giselind Adelmann, J. Nicholas P. Rawlins, Rolf Sprengel

**Affiliations:** ^1^Department of Experimental Psychology, University of Oxford, Oxford, United Kingdom; ^2^Departments of Molecular Neurobiology and Cell Physiology, Max Planck Institute for Medical Research, Heidelberg, Germany; ^3^EW-Nutrition GmbH, Visbek, Germany; ^4^Department of Physiology, Institute of Basic Medical Sciences, University of Oslo, Oslo, Norway; ^5^Department of Physiology and Pathophysiology, Heidelberg University, Heidelberg, Germany; ^6^OpenLab of Neurobiology, Kazan Federal University, Kazan, Russia; ^7^INSERM UMR 1249 Mediterranean Institute of Neurobiology (INMED), Aix-Marseille University Parc Scientifique de Luminy, Marseille, France; ^8^Esteve Pharmaceuticals, S.A., Barcelona, Spain; ^9^Institute of Anatomy and Cell Biology, University of Freiburg, Freiburg, Germany; ^10^Department for Internal Medicine, Klinikum Landkreis Tuttlingen, Tuttlingen, Germany; ^11^Institute for Anatomy and Cell Biology, Heidelberg University, Heidelberg, Germany

**Keywords:** AMPA receptors, GluA1, long-term potentiation, Morris water-maze, RNA-editing, spatial memory, spatial working memory

## Abstract

The GluA1 subunit of the L-α-amino-3-hydroxy-5-methyl-4-isoxazolepropionic acid receptor (AMPAR) plays a crucial, but highly selective, role in cognitive function. Here we analyzed AMPAR expression, AMPAR distribution and spatial learning in mice (*Gria1^R/R^*), expressing the “trafficking compromised” GluA1(Q600R) point mutation. Our analysis revealed somatic accumulation and reduction of GluA1(Q600R) and GluA2, but only slightly reduced CA1 synaptic localization in hippocampi of adult *Gria1^R/R^* mice. These immunohistological changes were accompanied by a strong reduction of somatic AMPAR currents in CA1, and a reduction of plasticity (short-term and long-term potentiation, STP and LTP, respectively) in the CA1 subfield following tetanic and theta-burst stimulation. Nevertheless, spatial reference memory acquisition in the Morris water-maze and on an appetitive Y-maze task was unaffected in *Gria1^R/R^* mice. In contrast, spatial working/short-term memory during both spontaneous and rewarded alternation tasks was dramatically impaired. These findings identify the GluA1(Q600R) mutation as a loss of function mutation that provides independent evidence for the selective role of GluA1 in the expression of short-term memory.

## Introduction

In the central nervous system (CNS) of adult mice L-α-amino-3-hydroxy-5-methyl-4-isoxazolepropionic acid receptors (AMPARs) are essential for fast synaptic transmission, hippocampal plasticity and behavioral performance. The core of the AMPAR complex in mature excitatory neurons is a hetero-tetrameric ion-channel consisting of combinations of three different subunits (GluA1–3), each encoded by a separate gene (*Gria1–3*; Keinanen et al., 1990). A fourth subunit, GluA4, is expressed mainly in the cerebellum, olfactory bulb and in interneurons, and only transiently during development in CA1 pyramidal neurons. GluA4 is not involved in AMPAR-mediated signal transmission in excitatory neurons of adult mice (Monyer et al., 1991; Zhu et al., 2000; Pelkey et al., 2016; Luchkina et al., 2017).

Studies with AMPAR gene knock-out mice identified the GluA1 subunit as an essential subunit for long-term potentiation (LTP) at hippocampal synapses and the (Q/R) site edited GluA2 subunit (Sommer et al., 1991; Burnashev et al., 1992) as the crucial AMPAR subunit for Ca^2+^-impermeable AMPARs. GluA1 knock-out mice (*Gria1^−/−^*) appear indistinguishable from their wild-type littermates by visual inspection in their home cage environment (Bannerman et al., 2004) and on tests for spatial reference memory, but they exhibit a robust and enduring spatial working/short-term memory deficit (Reisel et al., 2002; Schmitt et al., 2003; Sanderson et al., 2010). In contrast, GluA2 deficient mice (*Gria2^−/−^*) mice suffer from major behavioral impairments, including reduced exploration and profoundly impaired motor coordination, and therefore are not suitable for behavioral analysis of cognitive functions (Jia et al., 1996; Gerlai et al., 1998). Furthermore, (Q/R) site-editing deficient *Gria2^ΔECS^* mice die from epileptic seizures during adolescence (Brusa et al., 1995) but survive when the edited glutamine codon (CAG; Q) was replaced by an arginine codon (CGG; R; GluA2(Q607R)) in gene targeted mice (Kask et al., 1998; Higuchi et al., 2000).

The functional importance of a homologous mutation (Q600R) in the GluA1 subunit has not yet been investigated, despite the fact that: (i) in heterologous systems the GluA1(Q600R) subunit leads to the accumulation of GluA1(Q600R) assemblies in the endoplasmic reticulum (ER) and inhibits the formation and efficient membrane insertion of GluA1(Q600R) homomers, similar to tetrameric AMPAR assemblies with more than two GluA2(R607) subunits (Greger et al., 2002, 2003); and that (ii) gene-targeted mice encoding GluA1(Q600R) (*Gria1^tm1Erk^*; *Gria1^R/R^*) exhibit regular basic behaviors and reflexes, and do not suffer from developmental disorders (Vekovischeva et al., 2001, 2004). To study the molecular, physiological and behavioral effects of GluA1(Q600R) point mutation in detail, we have now analyzed AMPAR expression, synaptic function and learning behavior of *Gria1^R/R^* mice.

## Materials and Methods

### Ethical Statement

Animals for molecular and histological experiments were killed under the protocol MPI/T-6/06; 15/08; 20/9; and 28/11, according to the guidelines of the Max Planck Institute that adhere to ethical guidelines set by the local governing body and were performed according to the German Animal Welfare Act: Regulation for the Protection of Animals Used for Experimental or Other Scientific Purposes (Animal Welfare Regulation Governing Experimental Animals (TierSchVersV). Electrophysiological experiments were conducted according to the Norwegian Animal Welfare Act and the European Union’s Directive 86/609/EEC. All behavioral experiments were conducted in accordance with the United Kingdom Animals Scientific Procedures Act (1986), under the project license number PPLs 30/1505 and 30/1989 of the UK Home Office and by the license of the regional council in Karlsruhe, Germany (35-9185.81/G-71/10). Genetic manipulations of mice were performed under the license of the regional council in Karlsruhe, Germany (35-9185.81/G-4/02). Efforts were made to minimize the number of animals used.

### Mice

*Gria1^R/R^* (*Gria1^tm1Erk^* MGI:2178080) and *Gria1^−/−^* mice (*Gria1^tm1rsp^*; MGI:2178057) were generated by analogous targeting vectors and the same targeting strategy (Zamanillo et al., 1999; Vekovischeva et al., 2001) using R1 mouse embryonic stem cells (Nagy et al., 1993). Both targeting vectors for ES gene replacement of *Gria1^R/R^* and *Gria1^−/−^* just differed in the amino acid codon R600 and Q600 in the floxed exon 11, respectively. For the generation of *Gria1^−/−^* mice exon 11 was removed together with the NEO selection marker by Cre-recombinase mediated deletion. For the generation of *Gria1^R/R^* mice the floxed exon 11, encoding (Q600R) exon 11 is still present and only the floxed selection marker in intron 11 was removed by Cre. Similarly, in *Gria1^f/f^* mice (MGI:3798452; Jackson labs: 019012-B6N129-*Gria1^tm2Rsp/J^*) the floxed exon 11, encoding (Q600) is still present and expressed at regular levels (Fuchs et al., 2007). Cohorts of aged-matched *Gria1^R/R^* and *Gria1^+/+^ (WT)* littermates were produced by heterozygous matings, yielding approximately 25% *WT* and 25% *Gria1^R/R^* mice. The GluA1(Q600R) mutation was previously called GluA1(Q582R) and is now renumbered according to the full-length GluA1 precursor (Genbank: NM_001113325). *Gria1^−/−^* mice (MGI:2178057; Zamanillo et al., 1999) are available from the Jackson Laboratories (*Gria1^tm1rsp^*, stock number: 019011). All behavioral experiments were performed during the light phase.

### Antibodies

Anti-GluA1 (RRID:AB_390157), anti-GluA2 (RRID: AB_2336198), anti-GluA2/3 (RRID:AB_11213931), anti-GluA3 (RRID:AB_11152621), anti-GluN1 (RRID:AB_390129), anti-CaMKII (RRID:AB_2067919), anti-ß-actin (RRID: AB_476692), anti-mouse (RRID:AB_2336176), anti-rabbit (RRID:AB_2313567), gold-conjugated secondary antibodies (RRID:AB_106260) were used in these studies.

### Immunoblots

Hippocampi of mice were homogenized in 25 mM HEPES + Protease-Inhibitor Cocktail (Roche). Lysates were centrifuged for 5 min at 2000 rpm. Protein concentrations in supernatants were determined in triplets with BCA-Kit (Pierce). From each lysate 8 μg protein was separated by 7% SDS-PAGE and proteins were transferred to nitrocellulose membranes. The blotted proteins were probed with polyclonal antibodies against GluA1 (Merck Millipore 0.1 μg/ml), anti-GluA2 (Merck Millipore 0.16 μg/ml), anti-GluN1 (Merck Millipore, 0.25 μg/ml, monoclonal anti-GluA3 (ThermoFisher, 1:250), anti-CaMKII (MAB 8699 Merck Millipore 0.1 μg/ml) and anti-ß-actin (AC-15 Ascites, Sigma, 1:25,000), followed by peroxidase-linked anti-rabbit or -mouse secondary antibodies (Jackson Immuno Res., 1:20,000). ECL+ (ThermoFisher) was used to visualize immuno-labeled proteins.

### Immunoprecipitations (IPs)

Hippocampi of mice (>P60) were dissected and prepared as described above. Extracts were diluted 1:1 in buffer2 (25 mM HEPES pH7.4; 300 mM NaCl; 2% TritonX-100), incubated for 60 min at 4°C and subjected to centrifugation (10,000× *g* for 10 min at 4°C). Solubilized proteins of the supernatant (4 μg/μl) were mixed with buffer3 (25 mM HEPES pH7.4; 150 mM NaCl) in a 1:5 ratio. About 80 μg of solubilized protein was used for Immunoprecipitations (IPs) using protein A-Sepharose (ThermoFisher) washed in buffer4 (buffer2 and buffer3 in 1:5 ratio), and subsequently incubated for 1 h with the 80 μg of solubilized protein. About 15% of the supernatants were kept as “Input”. The rest of the supernatants were incubated overnight with anti-GluA1 or anti-GluA2/3 (1.5 μg; Merck Millipore), and then incubated for 1 h with 20 μl of the washed and pre-saturated protein A-sepharose. The A-sepharose was pelleted and kept as IP-pellet1. The supernatants were subjected to a second precipitation. The washed and pre-saturated protein A-sepharose beads were incubated with the specific antibodies for 1 h, washed with buffer4 after incubation with the supernatant for 4 h, pelleted (2 min; 3000× *g*), and pooled with pellet1. The pooled pellets were washed twice in buffer4 and buffer3 (800 μl each) and resuspended in 60 μl 0.5% SDS; 1% β-mercaptoethanol. After incubation at 95°C for 5 min the sample was briefly centrifuged and half of the sample was used for immunoblot analysis.

### Immunohistochemistry

At P14 and P42 mice were prepared for immunohistochemical analysis as described previously (Jensen et al., 2003). Fixed, agarose embedded brains were cut coronally into 50–75 μm vibratome sections (VT1000S, Leica Microsystems). For DAB-staining, sections were incubated for 10 min in 0.5% H_2_O_2_ in PBS, washed twice with PBS (10 min each) and blocked in 1% BSA, 0.3% TritonX-100, 2% NGS in PBS for 30–60 min. The sections were incubated at 4°C overnight in the same buffer containing the following primary antibodies: polyclonal anti-GluA1 (1 μg/ml, Merck Millipore), polyclonal anti-GluA2 (4 μg/ml, Merck Millipore), polyclonal anti-GluA2/3 (1 μg/ml, Merck Millipore,). After the incubation, sections were washed three times in buffer1 (0.3% BSA, 0.1% Triton X-100 in PBS) and exposed to horseradish peroxidase coupled secondary antibody (1:1000 anti-rabbit or -mouse, Jackson Immuno Research) in buffer1 for 2 h. Finally, sections were washed twice (10 min) with buffer1 followed by two washes with PBS. Sections were developed by incubation in 0.05% DAB solution (Sigma Aldrich) and 0.01% H_2_O_2_ in 20 mM Tris·Cl pH7.6. The reaction was stopped with 20 mM Tris·Cl pH7.6. The sections were mounted on glass slides, dried overnight and embedded *in vitro*-Clud (Langenbrinck Labor- und Medizintechnik).

### Microscopy

Bright-field images of immunostainings were captured with the Axio-Imager (Zeiss) and digitized with an AxioCam CCD camera using the AxioVision™ software. Brightness and color adjustments were performed in Photoshop CS5 (Adobe, RRID:SCR_014199). Confocal images of coronal brain slices immuno-stained by anti-GluA1 or GluA2 and Cy3 or FITC labeled secondary antibodies were recorded using a Leica TCS SP8 microscope (Leica).

### Quantification of Bright-Field and Confocal Images

Image data analysis was performed in ImageJ (V2.0.0.). Bright-field: the mean average signal intensity was measured for the corresponding ROIs in str. pyramidale, radiatum and oriens and was used to calculate the relative signal intensity in the str. pyramidale relative to the str. oriens + str. radiatum (Somatic Accumulation index SAi). For the confocal image analysis the mean signal intensity of big regions (at least 200 μm in length) covering the pyramidal cell layers and the neuropil in the str. radiatum were measured and used to calculate the somatic accumulation ratio.

### EM Immunocytochemistry

The preparation of the mouse brains was as described previously (Jensen et al., 2003). The fixed brains were cut on a vibratome into 300 μm sections. The CA1 area of the hippocampus was excised and the tissue blocks were cryoprotected in glycerol, cryofixed in nitrogen-cooled propane, and substituted in methanol-containing 1.5% uranyl acetate. After embedding in Lowicryl HM20 (Addivant Germany GmbH) ultrathin sections were processed for post embedding immunocytochemistry, employing anti-GluA1 and anti-GluA2 antibodies (5 μg protein/ml, Merck Millipore). Immunolabeling was visualized by 10 nm gold-coupled secondary antibodies (1:50, GE-Healthcare). Data analysis was performed on 12 grids per animal (six incubated with anti-GluA1; six with anti-GluA2). Sections from *WT* and *Gria1^R/R^* mice were processed simultaneously. On each grid, 300 randomly selected synapses were counted in CA1 str. radiatum. The number of labeled PSDs at contacts and the number of gold grains per individual PSD were evaluated. Only synapses labeled with at least two gold particles were counted. Data were fitted by Poisson distributions, and numbers of labeled PSDs and the mean numbers of gold grains per PSD were determined from the fitted distributions. Differences were assessed by Student’s *t*-test for paired samples.

#### Electrophysiology

##### Soma Patch Currents

For soma patch current analysis transverse hippocampal 250 μm slices were prepared from the brains of mice (P42–49) and recorded as described previously (Rozov et al., 2012). Whole-cell recordings from neurons were made at room temperature in voltage-clamp mode using a HEKA EPC-7 amplifier (Heka Electronik Dr. Schulze GMBH). Cells were held at −70 mV (Jensen et al., 2003). Currents were evoked by 2 ms, 1 mM glutamate application. AMPAR- and N-methyl-D-aspartate receptor (NMDAR)-mediated currents were isolated by treatment with 100 μM DL-AP5 (Sigma-Aldrich) and 10 μM NBQX (Tocris Cookson Ltd.), respectively. For statistical analysis, Student’s *t*-test was used, and data are presented as mean ± SD.

##### Field Recordings

For acute hippocampal slices mice (>P60) were killed with Suprane (Baxter Healthcare Corp.) and processed as described (Zamanillo et al., 1999; Jensen et al., 2003). Slices were recorded in an interface chamber exposed to humidified gas at 28–32°C and perfused with ACSF containing 2 mM CaCl_2_. To facilitate LTP induction we used 10 μM (–)-bicuculline methochloride (Tocris Cookson Ltd.) in some experiments. The resulting hyperexcitability was counteracted by increasing the concentrations of Ca^2+^ and Mg^2+^ up to 4 mM. In another set of experiments, 50 μM DL-AP5 (Sigma Aldrich) was added to the ACSF to block NMDAR-mediated synaptic plasticity.

For synaptic excitability and paired-pulse facilitation orthodromic synaptic stimuli (<300 μA, 0.1 Hz) were delivered through a tungsten electrode placed in str. radiatum, 100–150 μm from the pyramidal layer in the CA1 region. The presynaptic volley and the fEPSP were recorded by a glass electrode placed in str. radiatum. A second electrode placed in str. pyramidale monitored the population spike. Following a period of at least 20 min with stable responses, we stimulated the afferent fibers with increasing strength. A similar approach was used to elicit paired-pulse responses. The population spike amplitude was measured as the distance between the maximal population spike peak and a line joining the maximum pre- and postspike fEPSP positives. In order to pool data from the paired-pulse experiments, we selected responses to a stimulation strength just below the threshold for eliciting a population spike on the second fEPSP. For data analysis, data were pooled across mice of the same genotype and were statistically analyzed by linear mixed model analysis (SAS 9.1; SAS Institute Inc.), and presented as the mean ± SEM.

For LTP of synaptic transmission, orthodromic synaptic stimuli (50 μs, <280 μA, 0.2 Hz) were delivered alternately through two tungsten electrodes, one situated in the str. radiatum and another in the str. oriens of the hippocampal CA1 region. Extracellular synaptic responses were monitored by two glass electrodes (filled with ACSF) placed in the corresponding synaptic layers. After obtaining stable synaptic responses in both pathways (0.1 Hz stimulation) for at least 10–15 min, one of the following LTP induction paradigms was applied: (i) a single 100 Hz tetanization for 1 s; (ii) four such tetanizations given at 5 min intervals; or (iii) a theta-burst stimulation elicited by the stimulation electrode placed in str. radiatum with ten bursts, each consisting of six stimuli at 100 Hz, at intervals of 200 ms, repeated four times and 10 s apart, and paired with a simultaneous antidromic activation pattern elicited by a stimulation electrode placed in the alveus. To standardize across experiments, the stimulation strength used for tetanization of the str. radiatum synapses was just above the threshold for generation of a population spike in response to a single test stimulus. The synaptic efficacy was assessed by measuring the slope of the fEPSP in the middle third of its rising phase. Six consecutive responses (1 min) were averaged and normalized to the mean value recorded 1–4 min prior to tetanization. Data were pooled across animals of the same genotype, separately for the different LTP induction paradigms, and are presented as mean ± SEM. Statistical evaluation of LTP levels between tetanized and non-tetanized pathways were calculated by Student’s paired, two-tailed *t*-tests, whereas comparisons between genotypes and tetanization paradigms were statistically evaluated by linear mixed model analysis (SAS 9.1; SAS Institute Inc.).

#### Behavioral Phenotyping

Behavioral testing was carried out using *WT* (male, *n* = 8; female, *n* = 6) and *Gria1^R/R^* mice (male, *n* = 9; female, *n* = 6) of at least 4 months of age. Preliminary inspection and analysis of the data revealed that the sex of the mice had no effect on any of the behavioral tests (see “Results” section). For the purposes of data presentation, therefore, male and female mice were combined to give group sizes of 14 *WT* and 15 *Gria1^R/R^* mice (unless otherwise stated).

##### Spontaneous Locomotion

Spontaneous locomotor activity was assessed in a set of photocell activity cages. The mice were placed individually into the cages and activity was monitored for 2 h.

##### Spatial Working Memory

Spatial working memory (spontaneous alternation) was assessed in an enclosed, gray wooden T-maze (dimensions for goal arms: 30 cm long, 10 cm wide, 29 cm high). The entrances to the goal/choice arms contained sliding guillotine wooden doors. A central partition wall, extending 7 cm into the start arm, divided the choice point into two. The entrance to the choice point from the start arm also contained a wooden guillotine door. A light sprinkling of sawdust covered the floor of the maze. The mouse was placed into the start arm, facing the end wall, and was given a free choice of either goal arm. The sliding guillotine door of that arm was gently closed once the mouse had fully entered, and the mouse confined in that arm for 30 s. The central partition was then removed, all guillotine doors were opened, and the mouse replaced at the closed end of the start arm. Again the mouse had a free choice between the two goal arms. The number of alternations made by each mouse was recorded. Each mouse received 10 trials in total with a minimum inter-trial interval (ITI) of approximately 15 min.

Spatial working memory (non-matching to place testing) was also tested on an elevated wooden T-maze as described in detail (Deacon and Rawlins, 2006). Mice were maintained on a restricted feeding schedule at 85% of their free-feeding weight. A reward in the sample and the test run consisted of 0.1 ml of sweetened, condensed milk (diluted 1:1 with water). The time interval between the sample and choice run was 15 s. The animal was rewarded for choosing the previously unvisited arm. Mice were run one trial at a time with an ITI of at least 10 min. Mice received 30 trials in total.

##### Spatial Reference Memory

Spatial reference memory was assessed in the hidden platform Morris water-maze task (Morris, 1981; Morris et al., 1982), consisting of a large circular tank (diameter 2.0 m, depth 0.6 m), containing water at 20 + 1°C to a depth of 0.3 m. In order to escape from the water the mice had to find a fixed location, hidden escape platform (diameter 21 cm) submerged approximately 1 cm below the water surface. The water was made opaque by the addition of 2 l of milk. The pool was located in a well-lit laboratory containing prominent extramaze cues. Swim paths were monitored by video tracking. The mice received four trials per day (ITI = 15 s) for 9 days. The mice were placed into the pool facing the side wall at one of eight start locations (nominally N, S, E, W, NE, NW, SE and SW; chosen randomly across trials), and allowed to swim until they found the platform, or for a maximum of 90 s. On the 10th day of testing (24 h after spatial training trial 36), a probe trial was conducted. The platform was removed from the pool and the mice allowed to swim freely for 90 s. The percentage of time that animals spent in each quadrant of the maze was recorded.

Spatial reference memory was also examined using an elevated Y-maze made of black painted wood as described previously (Reisel et al., 2002; Bannerman et al., 2004). Mice received 10 trials per day with an ITI of 10 min. The number of correct choices was recorded. The maze was rotated by 120° randomly in either a clockwise or anticlockwise direction between each trial.

## Results

### The AMPAR Subunits GluA1(Q600R) and GluA2 Are Reduced in Hippocampi of *Gria1^R/R^* Mice but Are Still Involved in AMPAR Channel Assembly

**Figure 1:**
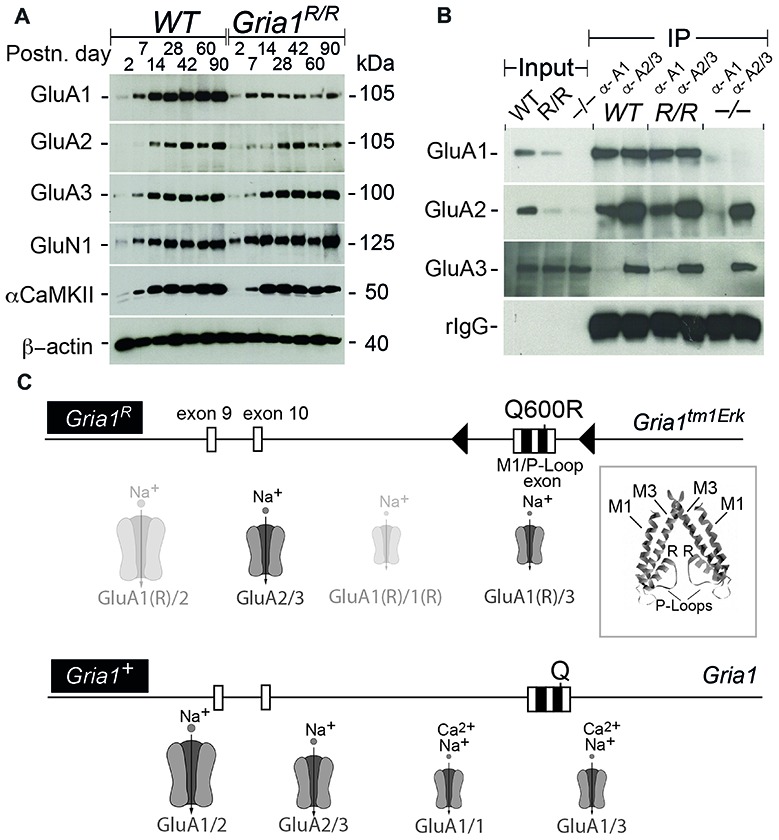
Expression of the L-α-amino-3-hydroxy-5-methyl-4-isoxazolepropionic acid receptor (AMPAR) subunits and subunit assemblies in *WT* and *Gria1^R/R^* mice. **(A)** Hippocampal expression of GluA1–3, GluN1, αCaMKII and ß-actin in *WT* and *Gria1^R/R^* mice from P2 till P90. **(B)** Co-immunoprecipitations (IPs) using polyclonal anti-GluA1 and anti-GluA2/3 antibodies show the presence of GluA1–3 in AMPAR assemblies from hippocampal membrane preparations at *P* > 60 of *WT*, *Gria1^R/R^* (R/R) and *Gria1^−/−^* (−/−) mice. **(C)** Schematic representation of the *Gria1^R^* “knock-in” (*Gria1^tm1Erk^*) allele and the *Gria1^+^* allele (*WT*). Below the gene segments, the putative AMPAR subtypes, that can operate at CA3-to-CA1 synapses in *Gria1^R/R^* and* WT* mice, are schematically depicted (GluA1(R) = GluA1(Q600R)). Large AMPAR symbols for high abundance; small symbols for low abundance; transparent for AMPARs with low single channel conductance. The inset shows the position of the Q600R mutations (R) in two out of the four P-loop segments that form the ion pore of an AMPAR. Exons are in boxes, loxP sites in black triangles and the M1 and P-loop coding sequence in black squares. The position of the mutated codon Q600R codon and codon Q600 in *Gria1^tm1Erk^* and *Gria1* are indicated, respectively (see Sprengel et al., 2001). High resolution images of **(A,B)** are accessible at https://dx.doi.org/10.17617/3.1i.

Since in heterologous systems the GluA1(Q600R) point mutation is trafficking compromised (Greger et al., 2003), and since hippocampal GluA2 expression levels are reduced in absence of GluA1 (Jensen et al., 2003), we first analyzed the expression and the channel assembly of AMPARs in *Gria1*^R/R^ mice by immunoblots and co-IPs. Our analysis of hippocampal protein extracts isolated at different postnatal days (P2 – P90) from controls and *Gria1^R/R^* mice showed that the dominant partner GluA2 and GluA1(Q600R) itself were reduced in *Gria1^R/R^* animals (Figure 1A). GluA1(Q600R) was reduced at all developmental stages. The reduction of GluA2 expression became obvious from P42 onwards. No changes were observed for the protein levels of GluA3, the NMDAR subunit GluN1 and for the alpha subunit of the Ca^2+^/Calmodulin dependent protein kinase 2 (α-CaMKII). Thus, the half-life of AMPARs containing GluA1(Q600R) seems to be reduced in adult *Gria1^R/R^* mice. The observed reduction of GluA2 was very similar to what we have described previously in *Gria1^−/−^* mice (Jensen et al., 2003).

For the analysis of the GluA1(Q600R) assemblies, we IP-purified GluA1- or GluA2/3-containing AMPARs from solubilized membrane preparations of hippocampi from *WT* controls and *Gria1^R/R^* mice. Comparable amounts of GluA2 could be co-purified with anti-GluA1 antibody, whereas very little GluA3 associated with GluA1 in *WT* mice and GluA1(Q600R) in *Gria1^R/R^* mice (Figure 1B). This is in line with the observation that heteromeric GluA1/A2 assemblies provide the majority of AMPAR assemblies in the hippocampus (Wenthold et al., 1996; Lu et al., 2009), and indicates that GluA1(Q600R) does form assemblies with GluA2. Similarly, anti-GluA2/3 antibodies co-precipitated GluA1 and GluA1(Q600R) from *WT* controls and *Gria1^R/R^* mice, respectively. The specificity of the IP was controlled by the crude membrane preparation of *Gria1^−/−^* mice, and IP input by visualizing immunoglobulin of the primary antibody. These results show that the GluA1(Q600R) subunit can form dimeric or tetrameric assemblies with GluA2 and GluA3 subunits indicating that the reduced levels of GluA1(Q600R) and GluA2 in *Gria1^R/R^* mice were not caused by a channel assembly impairment, but most likely reflect a shorter half-life of the low conductance AMPAR channels (Swanson et al., 1997) containing four subunits with R at the tip of the channel pore: GluA1(Q600R) and GluA2 homomeric and GluA1(Q600R)/GluA2 heteromeric channels (Figure 1C).

### Somatic Accumulation of GluA1(Q600R) and GluA2 in Principal Cells of the Hippocampus of *Gria1^R/R^* Mice

**Figure 2:**
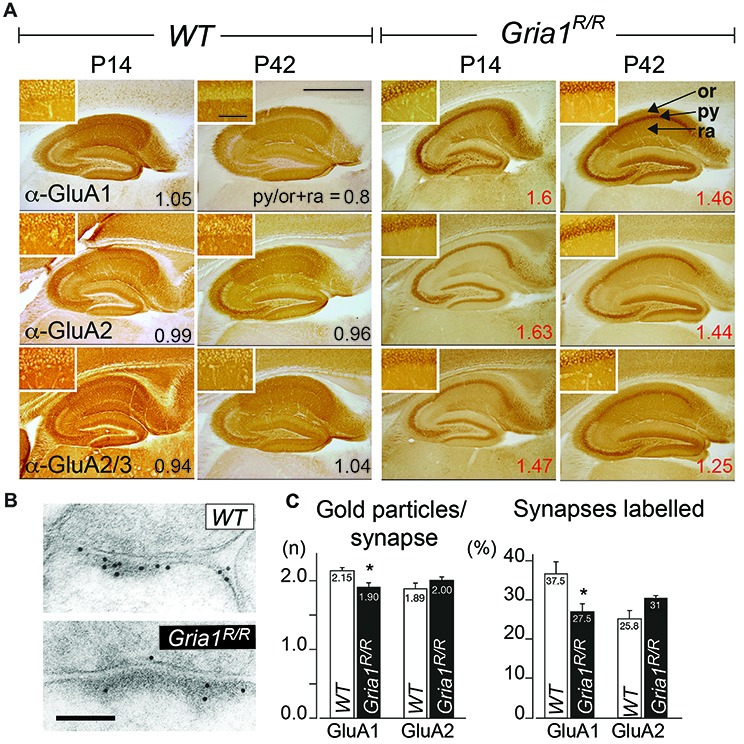
Subcellular AMPAR subunit distribution in the hippocampus. **(A)** Distribution of GluA1–3 subunits in hippocampi of *WT* controls and *Gria1^R/R^* mice at P14 and P42. The GluA1–3 subunit expression levels were detected by GluA1, GluA2 and GluA2/A3 subunit specific antibodies. The ratios of the average signal intensity in the str. pyramidale (py) vs. the signal intensity in the str. oriens (or) and radiatum (ra) are indicated (Somatic Accumulation index; SAi). Scale bar = 0.9 mm. Insets show higher magnifications of the respective str. pyramidale; scale bar = 50 μm. **(B)** Anti-GluA1 immunogold labeling of GluA1 and GluA1(Q600R) at an excitatory CA1 synapse taken from an adult *WT* and a *Gria1^R/R^* mouse, respectively. Scale bar = 0.1 μm. **(C)** Quantification of the immunogold signal at CA3-to-CA1 synapses containing GluA1, GluA1(Q600R) and GluA2 subunits. Intensity of GluA1- and GluA2-immunoreactivity expressed as number of gold particles per synapse. *Gria1^R/R^* mice showed significant fewer anti-GluA1 gold particles per synapse in comparison to *WT*. Moreover, the number of anti-GluA1 labeled synapses was significantly lower in *Gria1^R/R^* mice. Similar concentrations of anti-GluA2 gold particles were found in *Gria1^R/R^* and *WT* control mice; *n* = 6 *Gria1^R/R^* and 6 *WT* control mice. Error bars indicate SEM. Unpaired two-tailed student’s *t*-test was used (*P ≤ 0.05). For original data used for the quantification see https://dx.doi.org/10.17617/3.1i.

In the absence of GluA1, the GluA2 subunits accumulate in the cellular layers of hippocampal principle cells in young and in adult mice (Zamanillo et al., 1999; Jensen et al., 2003). To determine the expression pattern of GluA1(Q600R) and GluA2 in *Gria1^R/R^* mice we generated and evaluated immunostaining of dorsal hippocampi in coronal brain sections from young and adult GluA1(Q600R) expressing mice by using GluA1, GluA2 and GluA2/3 specific antibodies. As shown in Figure 2A, all three antibodies revealed an expression in all hippocampal layers in *WT* mice at P14 and P42. Hippocampi of *Gria1^R/R^* mice, however, exhibited strong accumulation of GluA1 and GluA2 immunosignal in principle cell layers of the hippocampus of *Gria1^R/R^* mice at P14 and P42 similar to the GluA2 accumulation, which can be observed in *Gria1^−/−^* mice (Jensen et al., 2003). To quantify the subcellular distribution of the AMPAR subunits we calculated somatic accumulation indices (SAi) in the CA1 region showing the highest AMPAR expression in the hippocampus. In *Gria1^R/R^* mice ratios of mean signal intensities in the principle cell (str. pyramidale) vs. dendritic layers (str. oriens, str. radiatum) above one (SAi = 1.39–2.14) indicated strong somatic accumulation of the GluA1, A2 and A2/A3 imunosignal at P14 and at P42 whereas AMPAR subunits in *WT* mice showed no clear hippocampal layer specific preference, with a SAi of approximately one. Confocal fluorescent images of anti-GluA1 and anti-GluA2 immunostainings confirmed the somatic accumulation of GluA1 and GluA2 in the hippocampus of *Gria1^R/R^* mice (Supplementary Figure S1). To analyze the effect of the GluA1(Q600R) mutation on synaptic receptors directly we performed a quantitative electron microscopic immunogold analysis for GluA1 and GluA2 subunits at CA3-to-CA1 PSD containing synapses (Figure 2B). As shown in Figure 2C, there was no significant difference in the number of postsynaptic anti-GluA1, GluA1(Q600R) and anti-GluA2 immunosignals between *WT* controls and *Gria1^R/R^* mice. Furthermore, we were unable to detect any statistically significant difference between *Gria1^R/R^* and *WT* mice in the number of immunoreactive GluA1 and GluA2 subunits per synapse. A slight but significant reduction in the number of synapses expressing GluA1(Q600R) could, however, be detected in *Gria1^R/R^* mice when compared to the GluA1 expression in *WT* littermates (Figure 2C). The possibility of a direct loss of extrasynaptic AMPARs could not be analyzed by our current immunogold analysis and would require cryosection immunogold analysis.

Thus, the replacement of GluA1 by the mutated GluA1(Q600R) had no substantial effect on the synaptic presence of GluA1(Q600R) and GluA2 subunits but induced a retention of GluA1(Q600R) and GluA2 at somata of principal neurons of *Gria1^R/R^* mice.

### Reduced Somatic Currents in *Gria1^R/R^* Mice

**Figure 3:**
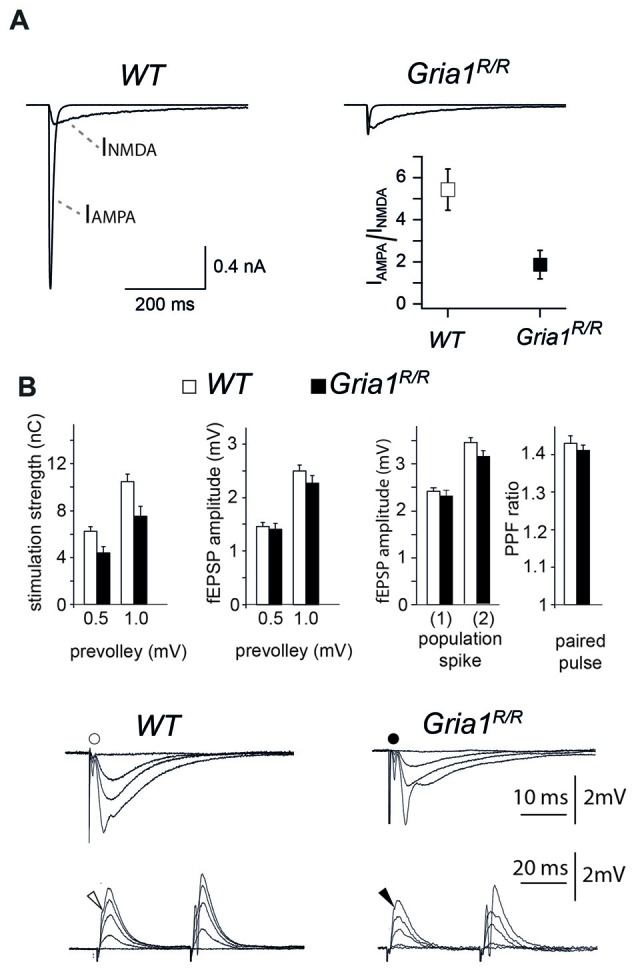
Whole-soma currents, excitatory synaptic transmission, synaptic excitability and paired-pulse facilitation in the CA1 hippocampal region. **(A)** Recordings of glutamate activated AMPAR and *N*-methyl-D-aspartate receptor (NMDAR) currents from nucleated patches of CA1 pyramidal cells obtained from P42 *WT* (left) and *Gria1^R/R^* (right) mice. I_AMPAR/NMDAR_ ratio is significantly different at P42; I_AMPAR/NMDAR_ ratio 1.87 ± 0.68 in *Gria1^R/R^*, *n* = 7 from 3 mice; 5.43 ± 0.97 in *WT*, *n* = 9 from 4 mice; *p* < 0.01. **(B)** Left: stimulation strengths (in nC) necessary to elicit a prevolley of a given amplitude (0.5 mV, 1.0 mV and 1.5 mV) in slices from *WT* (open columns) and *Gria1^R/R^* (filled columns) mice. Middle left: fEPSP amplitudes in the two genotypes as a function of the three prevolley amplitudes. Middle right: (1) the fEPSP amplitudes in slices from the two genotypes necessary to elicit a just detectable population spike; and (2) a population spike of 2 mV amplitude. Right: paired-pulse facilitation (PPF) ratio in the two genotypes at an inter-stimulus interval of 50 ms. Lower panels: each trace is the mean of five consecutive synaptic responses in str. radiatum elicited by different stimulation strengths in slices from *WT* (left) and *Gria1^R/R^* (right) mice. The prevolleys preceding the fEPSPs are indicated by circles. The lower recordings show traces from str. pyramidale elicited by paired-pulse stimulation (50 ms interstimulus interval). Arrowheads indicate the population spike threshold. The number of experiments ranged from 30 to 82. Data are shown as mean + SEM.

In GluA1 deficient mice somatic redistribution of functional AMPARs in CA1 cells could be documented by loss of somatic and dendritic AMPAR responses, slightly reduced synaptic efficiency and impaired LTP (Zamanillo et al., 1999; Hoffman et al., 2002; Andrásfalvy et al., 2003; Jensen et al., 2003). To provide electrophysiological evidence that the expression of the GluA1(Q600R) mutation has a similar electrophysiological signature, we first monitored the somatic AMPAR/NMDAR ratios by analyzing glutamate-induced currents in nucleated patches. We found that, similar to GluA1 deficient mice, the glutamate-induced AMPAR currents were strongly reduced in nucleated patches of *Gria1^R/R^* mice at P42, and reached, at most, only 20% of the *WT* control values (AMPAR/NMDAR ratios; 1.87 ± 0.68 in *Gria1^R/R^*, *n* = 7 cells from three mice; 5.43 ± 0.97 in *WT*, *n* = 9 cells from 4 mice; *p* < 0.01; Figure 3A). To assess changes in excitatory synaptic transmission and synaptic excitability, we recorded simultaneously in CA1 apical dendritic (middle part of the str. radiatum) and soma layers of hippocampal slices from *Gria1^R/R^* and* WT* littermates. We measured the fiber volley, the fEPSP, and the population spike as a function of different stimulation strengths. The stimulation strengths necessary to elicit fiber volleys of given amplitudes (0.5 and 1.0 mV) were numerically lower in *Gria1^R/R^* (4.4 ± 0.5 nC, *n* = 44; 7.5 ± 0.9 nC, *n* = 44) compared to control *WT* mice (6.2 ± 0.4 nC, *n* = 82; 10.4 ± 0.7 nC, *n* = 80), although this didn’t reach statistical significance *p* = 0.08 and 0.06, respectively (Figure 3B). The evoked fEPSPs for presynaptic fiber volleys of 0.5 and 1.0 mV in *Gria1^R/R^* mice (1.4 ± 0.1 mV, *n* = 44 and 2.3 ± 0.1 mV, *n* = 44), were not significantly different from the fEPSPs elicited in brain slices of control littermates (1.5 ± 0.1 mV, *n* = 82; 2.5 ± 0.1 mV, *n* = 80, *p* = 0.70 and 0.41, respectively), indicating that basal, fast glutamatergic transmission was unaltered in *Gria1*^R/R^ mice, at least across this range of stimulation intensities. Furthermore, postsynaptic excitability measured as the fEPSP threshold for generating a just detectable population spike was unchanged in *Gria1^R/R^* mice (2.3 ± 0.1 mV, *n* = 44), when compared to controls (2.4 ± 0.1 mV, *n* = 83) *p* = 0.58 (Figure 3B). In a similar manner, the fEPSP size necessary to elicit a population spike of 2 mV was also of the same magnitude in the two genotypes (*Gria1^R/R^* 3.1 ± 0.1 mV, *n* = 40;* WT* 3.4 ± 0.1 mV, *n* = 73 *p* = 0.24). A comparison of paired-pulse facilitation (PPF ratio) also failed to reveal a significant difference between *Gria1^R/R^* (1.41 ± 0.02 mV, *n* = 48) and *WT* littermates (1.43 ± 0.02 mV, *n* = 44 *p* = 0.50; Figure 3B). Thus similar to GluA1-deficient mice, the somatic AMPAR currents could hardly be detected despite regular synaptic transmission in field recordings in hippocampal CA1 cells of *Gria1^R/R^* mice.

### Loss of Synaptic Plasticity at CA3-to-CA1 Synapses in *Gria1^R/R^* Mice

**Figure 4:**
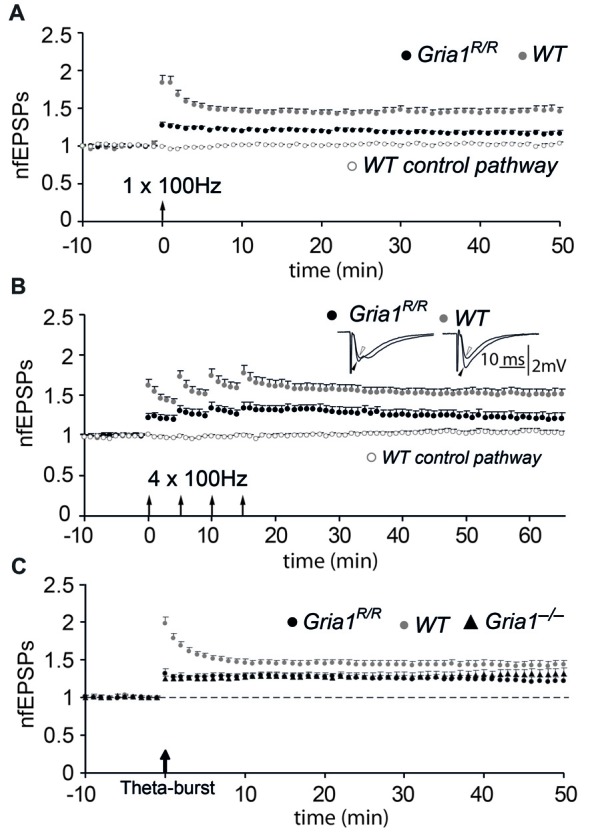
Diminished long-term potentiation (LTP) in *Gria1^R/R^* mice. **(A)** Normalized and pooled fEPSP slopes evoked in str. radiatum at CA3-to-CA1 synapses in slices from *Gria1^R/R^* and *WT* mice. LTP was induced by a single tetanization. For the sake of clarity, only the non-tetanized control pathway in control *WT* mice is shown. Arrow at the abscissa indicates the time of tetanic stimulation. Vertical bars indicate SEM.** (B)** As in **(A)**, but four tetanizations were used for LTP induction. Insets show means of six consecutive synaptic responses in the tetanized pathway before (open arrowhead) and 45 min after (filled arrowhead) tetanization in an experiment from a *Gria1^R/R^* (left sweeps) and a *WT* (right sweeps) control.** (C)** As in **(A)**, but LTP was induced by a theta-burst paradigm where synaptically and antidromically evoked responses were paired. For comparison, experiments performed in slices from the brains of *Gria1^−/−^* mice are also shown.

The loss of synaptic plasticity at hippocampal CA3-to-CA1 synapses in the presence of synaptic AMPAR mediated transmission was the most significant result provided by the *Gria1* knock-out mice and is an additional signature for the loss of GluA1. Therefore we examined LTP at hippocampal CA3-to-CA1 synapses in brain slices from adult mice. Forty to 45 min after a single tetanization (100 Hz, 1 s) of the afferent fibers in the str. radiatum or str. oriens in slices from control mice, the average slope of the field EPSP was 1.44 ± 0.05 (mean ± SEM; *n* = 26) of the pre-tetanic value, whereas the untetanized control pathway was unchanged (1.02 ± 0.02). We found a significant reduction in LTP in slices from *Gria1^R/R^* mice (1.17 ± 0.03, *n* = 25, when compared to *WT*; *p* < 0.01; Figure 4A), although this reduction in the magnitude of LTP was not as dramatic as observed previously in *Gria1^−/−^* mice (Zamanillo et al., 1999). To ascertain whether the reduced LTP in the *Gria1^R/R^* mice was caused by an induction failure, such as a change in the LTP induction threshold, we conducted a further series of experiments in the presence of the GABA_A_-receptor blocker (–)-bicuculline methochloride (10 μM), which facilitates the induction of LTP without significantly changing its magnitude (Wigström and Gustafsson, 1983, 1985). Similar to the previous results with *Gria1^−/−^* mice (Zamanillo et al., 1999), the evoked LTP under these circumstances was not significantly different from the values obtained in the control solution, either in *WT* mice (1.33 ± 0.04, *n* = 37, *p* = 0.10) or in *Gria1^R/R^* mice (1.07 ± 0.04, *n* = 26, *p* = 0.08), suggesting that the tetanization procedure used previously was sufficient. LTP was also reduced in *Gria1^R/R^* mice when a repeated tetanization paradigm was applied (100 Hz for 1 s, repeated 4× with 5 min interval). LTP induced by this procedure in CA1 slices from *WT* controls was 1.51 ± 0.06 (*n* = 17), whereas in *Gria1^R/R^* mice LTP measured 1.22 ± 0.06 (*n* = 15; Figure 4B; genotype comparison *p* < 0.01). The residual LTP in *Gria1^R/R^* mice was NMDAR-mediated. It was completely blocked by the presence of 50 μM AP5 (data not shown), as previously also observed in *Gria1^−/−^* mice (Jensen et al., 2003). Notably, despite this pronounced deficit in tetanus-induced LTP (Zamanillo et al., 1999), more recent studies have shown that a theta-burst pairing paradigm (Larson and Lynch, 1986) can elicit robust LTP in *Gria1^−/−^* mice (Hoffman et al., 2002; Romberg et al., 2009). We therefore adopted a similar theta-burst pairing paradigm, but used extra-cellular antidromic activation instead of depolarizing soma injection in order to elicit a single postsynaptic spike (Figure 4C). In control mice, the magnitude of LTP induced with the theta-burst pairing paradigm was not significantly different from the magnitude obtained by the tetanization procedures (1.43 ± 0.06, *n* = 30). Furthermore, although reduced in magnitude, the amount of LTP induced using the theta-burst stimulation paradigm in both full knockouts and point mutants was not significantly different from that observed in the *WT* control mice (*Gria1^R/R^*: 1.23 ± 0.05, *n* = 14; *Gria1^−/−^*: 1.30 ± 0.06, *n* = 17; *p* = 0.15 and *p* = 0.21, respectively, when compared to *WT*). However, in agreement with our previous observations in *Gria1^−/−^* mice (Zamanillo et al., 1999; Hoffman et al., 2002; Jensen et al., 2003), the potentiated fEPSP responses during the first few minutes after tetanization were reduced in *Gria1^R/R^* mice. In *WT* animals this immediate increase in synaptic transmission comprises post-tetanic potentiation (PTP), short-term potentiation (STP) and LTP (Bliss and Lomo, 1973). This immediate component of synaptic plasticity was significantly reduced (*p* < 0.05) during the first 7–10 min following LTP induction in both *Gria1^−/−^* and *Gria1*^R/R^ mice and thus might be dependent on the presence of Ca^2+^-permeable GluA1-containing AMPA receptors that are absent in *Gria1^−/−^* and *Gria1^R/R^* mice (Rozov et al., 2012).

### Selective Spatial Working Memory Impairment in *Gria1^R/R^* Mutant Mice

Given that the changes in AMPAR expression and AMPAR mediated synaptic plasticity are comparable between *Gria1^−/−^* and *Gria1^R/R^* mice, we hypothesized that *Gria1^−/−^* and *Gria1^R/R^* mice would show similar behavioral impairments. Most notably, *Gria1^−/−^* mice exhibit locomotor hyperactivity in a novel environment, and a profound and selective spatial short-term memory deficit (Reisel et al., 2002; Schmitt et al., 2003; Bannerman et al., 2004). In agreement with this, the *Gria1^R/R^* mice displayed a pronounced hyperactivity in photocell locomotor activity cages. ANOVA revealed a significant main effect of genotype (Table 1). There was no main effect of sex, or any interaction between sex and genotype (*F* < 1 for both).

**Table 1:** Effects of GluA1(Q600R) on locomotor activity and spatial working memory during the spontaneous alternation T-maze test.

Task/Measure	*WT* Male	*WT* Female	*Gria1^R/R^* Male	*Gria1^R/R^* Female	Statistics (effect of genotype)
Spontaneous locomotor activity					
Total beam breaks (2 h)	2861 ± 419	2355 ± 295	4246 ± 431	4572 ± 439	*F*_(1,25)_ = 18.0; *p* < 0.01
Spontaneous alternation					
% alternation	87.5 ± 3.1	71.7 ± 7.0	61.1 ± 5.9	43.3 ± 8.0	*F*_(1,25)_ = 20.4; *p* < 0.01

*WT littermates (n = 14) and *Gria1^R/R^* mice (n = 15). Data are presented as means (± SEM) and were analyzed by analysis of variance. Where necessary data were subjected to log_10_ transformation prior to analysis*.

*Gria1^R/R^* mice also displayed a robust and selective spatial short-term memory deficit. In the discrete trial, spontaneous alternation, spatial working memory task the *WT* controls showed a high level of alternation performance across the 10 trials (80.7%), whereas the *Gria1^R/R^* mice exhibited chance levels (54.0%; Table 1), thus resembling the result seen with *Gria1^−/−^* mutants (Bannerman et al., 2004; Sanderson et al., 2007). An ANOVA revealed a significant effect of genotype (Table 1), and a significant effect of sex that was due to an overall lower level of alternation in the females (*F*_(1,25)_ = 7.68; *p* < 0.05). There was, however, no interaction between genotype and sex (*F* < 1; *p* > 0.20).

**Figure 5:**
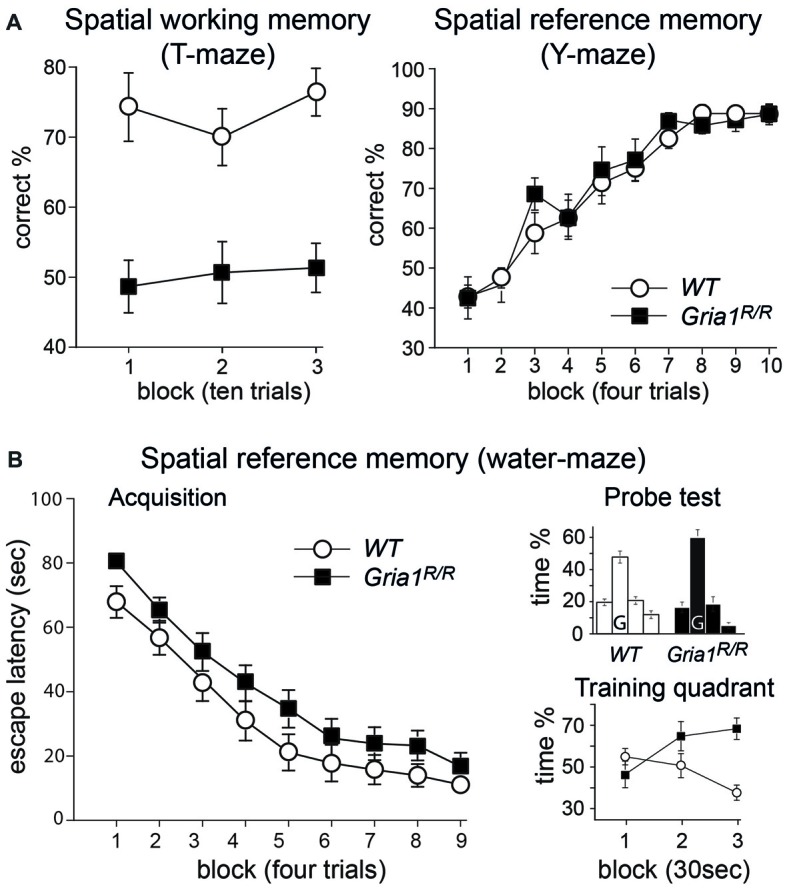
Spatial memory in *Gria1^R/R^* mice. **(A)** Left: *Gria1^R/R^* mice are impaired on a spatial working memory task on the elevated T-maze. Mean percentage correct responses (± SEM) for *WT* littermates (white circles; *n* = 14) and *Gria1^R/R^* mice (black squares; *n* = 15) during spatial non-matching to place testing on the elevated T-maze. Right: *Gria1^R/R^* mice show normal spatial reference memory acquisition on the elevated Y-maze. Mean percentage of correct responses (± SEM) during acquisition of an appetitive spatial reference memory task for male *WT* littermates (white circles; *n* = 8) and *Gria1^R/R^* mice (black squares; *n* = 7). **(B)** Left: *Gria1^R/R^* mice acquired a standard spatial reference memory version of the Morris water-maze task. Mean escape latency (± SEM) for each day of testing during acquisition for *WT* littermates (white circles; *n* = 14) and *Gria1^R/R^* mice (black squares, *n* = 15). Right, top: mean percentage of time (± SEM) spent in the four quadrants of the pool during the 90 s probe test, conducted at the end of spatial training (after 36 trials) for *WT* littermates (left; *n* = 14) and *Gria1^R/R^* mice (right; *n* = 15). The platform had previously been located in the training quadrant (G) during acquisition. Right, bottom: mean percentage of time (± SEM) spent in the training quadrant during the three 30 s time blocks of the 90 s probe trial by *WT* littermates (white circles; *n* = 14) and *Gria1^R/R^* mice (black squares, *n* = 15).

*Gria1^R/R^* mice also demonstrated a profound spatial short-term memory impairment during the appetitively motivated, discrete trial, rewarded alternation task on the elevated T-maze (Figure 5A, left). Whereas the *WT* controls performed well on the task (74.2 ± 2.3% correct responses), the *Gria1^R/R^* mice never differed from chance levels of performance (50.4 ± 2.3%). An ANOVA confirmed that there was a significant effect of genotype (*F*_(1,25)_ = 54.09; *p* < 0.01). There was no effect of sex, nor a genotype by sex interaction (*F*’s < 2.77; *p*’s > 0.10 for both).

In contrast to these spatial working memory tasks, *Gria1^R/R^* mice performed normally on an appetitively motivated, spatial reference memory task on the elevated Y-maze (Figure 5A, right). Previous studies have demonstrated that *Gria1^−/−^* mice acquire this task as well as *WT* controls (Reisel et al., 2002). In the present study, both *WT* and *Gria1^R/R^* mice learned the appetitive, spatial reference memory task at a similar rate, and reached the same high level of asymptotic performance (Figure 5A, right). An ANOVA of the percent correct responses across the 10 days of testing confirmed these impressions. There was a significant effect of day (*F*_(9,117)_ = 41.68; *p* < 0.01), but no significant effect of genotype, nor any genotype by day interaction (*F* < 1 for both).

Similarly, *Gria1^R/R^* mice also performed well on the spatial reference memory version of the Morris water-maze task (Figure 5B), again paralleling the results that have previously been obtained for *Gria1^−/−^* mice (Zamanillo et al., 1999; Reisel et al., 2002). Both groups of mice became gradually more efficient at reaching the escape platform, as shown by the gradual reduction in escape latencies as training proceeded (main effect of day—*F*_(8,200)_ = 62.32; *p* < 0.01; Figure 5B, *left*). Although the mutant mice appeared to take slightly longer than their *WT* counterparts to find the escape platform throughout training, this was not reflected in a statistically significant main effect of genotype (*F*_(1,25)_ = 3.55; *p* > 0.05). Furthermore, there was no interaction between genotype and day (*F* < 1). In addition, the ANOVA revealed that there was no significant effect of sex (*F*_(1,25)_ = 1.77; *p* > 0.10), and sex did not interact with any of the other experimental variables (*F* < 1 for all interaction terms). Analysis of path lengths to the escape platform revealed a similar pattern of results. There was a highly significant main effect of day, reflecting the fact that the mice learned to swim more directly towards the location of the hidden platform (*F*_(8,200)_ = 53.08; *p* < 0.01). Again, although the *Gria1^R/R^* mice traveled slightly further before finding the platform, this did not result in a significant main effect of genotype (*F*_(1,25)_ = 3.21; *p* > 0.05). Furthermore, there was no genotype by day interaction (*F*_(8,200)_ = 1.32; *p* > 0.20). As before, the ANOVA also showed no main effect of sex, nor any interaction between sex and the other experimental variables (*p* > 0.10 for all interaction terms).

The performance during the 90 s probe trial conducted at the end of water-maze training confirmed that both the *WT* and *Gria1^R/R^* mice had learned about the spatial location of the escape platform, with both groups showing a strong preference for the training quadrant (Figure 5B, right top). ANOVA comparing the time spent in each of the four quadrants of the pool during the probe trial revealed a significant effect of quadrant (*F*_(2,75)_ = 47.79; *p* < 0.01), reflecting the fact that both groups of mice showed a strong preference towards the training quadrant (% time in the training quadrant during the 90 s probe trial—*WT* = 47.7; *Gria1^R/R^* = 59.9). Note that for the analysis of the distribution of time spent searching the four quadrants, the numerator term in the degrees of freedom was reduced by one to control for the fact that the quadrant dwell times were not independent. There was no interaction between quadrant and genotype, quadrant and sex, nor quadrant by genotype by sex (*p* > 0.10 for all interactions). A second ANOVA was then conducted comparing only the amount of time spent in the training quadrant. For this analysis the 90 s probe trial was divided into three 30 s time bins (Figure 5B, right bottom). This ANOVA confirmed that the overall time spent in the training quadrant during the 90 s probe trial by the two groups did not differ; although the mutant mice spent slightly more time in the training quadrant, this tendency did not attain statistical significance (*F*_(1,25)_ = 2.92; *p* > 0.05). ANOVA revealed no significant effect of sex or time bin (*p* > 0.20 for both comparisons), no genotype by sex, sex by time bin, or genotype by sex by time bin interaction (*p* > 0.10 for all interactions). There was, however, a statistically significant genotype by time bin interaction (*F*_(2,50)_ = 10.20; *p* < 0.01), which reflected the fact that as the probe trial proceeded, the *Gria1^R/R^* mice were spending more time in the training quadrant relative to the *WT* controls. This was confirmed by a subsequent analysis of simple main effects which showed that there were no significant group differences during either the 0–30 or 30–60 s time bins of the probe trial (*p* > 0.10 for both comparisons), but that there was a significant difference between the groups in terms of the amount of time spent in the training quadrant during the final 30 s of the transfer test (60–90 s *F*_(1,25)_ = 22.17; *p* < 0.01).

One possible explanation as to why the *Gria1^R/R^* mice might have remained in the training quadrant to a greater extent than the *WT* mice as the probe test proceeded is that they exhibited increased floating behavior, having discovered that the platform was not present. However, only two mice showed any signs of floating behavior during the probe test. Both were male *Gria1^R/R^* mice, and they each spent approximately 10 s floating during the 90 s test. Re-analysis of the probe test data for the time spent in the training quadrant only, excluding these two subjects, revealed essentially the same pattern of results, although now the overall main effect of genotype did reach statistical significance (*F*_(1,23)_ = 10.94; *p* < 0.01), with the point mutants spending significantly more time in the goal quadrant during the probe test. Importantly, there was still a genotype by time bin interaction (*F*_(2,46)_ = 10.20; *p* < 0.01), and subsequent analysis of simple main effects again showed that the stronger preference for the training quadrant in the point mutants only developed as the probe test proceeded (main effect of genotype for 0–30 s *F* < 1; 30–60 s *F*_(1,23)_ = 4.55; *p* < 0.05; 60–90 s *F*_(1,23)_ = 43.36; *p* < 0.01).

To ensure that the pattern of results that we obtained across the spatial memory tests did not reflect the order of testing, we re-tested the mice on the discrete trial, rewarded alternation test after they had completed the spatial reference memory tasks. As before, the *WT* animals again showed a good level of spatial short-term memory performance (85.6%), whereas the *Gria1^R/R^* mice performed at near chance levels (45.0%; *F*_(1,13)_ = 62.98; *p* < 0.01). The results were therefore very similar to the earlier assessments of spatial short-term memory during both spontaneous and rewarded alternation, and demonstrate that the pattern of positive and negative results in the preceding tasks was not due to the order of testing. Thus, in summary, *Gria1^R/R^* mice exhibited an impairment on spatial working/short-term memory tasks but preserved spatial reference memory acquisition, both in the appetitively motivated Y-maze task and in the Morris water-maze.

## Discussion

*Gria1^R/R^* mice with gene-targeted mutagenesis of the *Gria1* gene, in which the amino acid codon for glutamine (Q600) was replaced by an arginine codon (R600), showed reduced hippocampal expression levels and somatic accumulation of GluA1(Q600R) and GluA2 subunits in CA1, CA3 and DG granular cells. *Gria1^R/R^* mice exhibited reduced synaptic strengthening following tetanic stimulation at CA3-to-CA1 synapses and a slight reduction of CA1 AMPARs and AMPAR currents. This pattern of results is virtually identical to what has been observed previously in *Gria1^−/−^* mice (Zamanillo et al., 1999).

The somatic accumulation of AMPARs in hippocampal principal cells of *Gria1^R/R^* mice can be explained by the high proportion of AMPAR subunits that contain a P-loop arginine amino acid residue (Q/R site edited GluA2 and GluA1(Q600R)) in *Gria1^R/R^* mice. In *WT* mice, the expression of the Q/R site edited GluA2 subunits (Sommer et al., 1991) is well balanced by GluA1 and GluA3 levels since GluA2 subunit—containing Q/R site edited R607—can build preferentially heteromeric channel assemblies with GluA1 and GluA3, containing Q600 at the homologous position (Greger et al., 2002, 2003). Because the GluA3 subunit is not strongly expressed in the hippocampus, the majority of AMPARs are composed of GluA1 and Q/R-site edited GluA2 (Wenthold et al., 1996; Lu et al., 2009). However, in *Gria1^R/R^* mice the mutation of GluA1 into GluA1(Q600R) leads to a dramatic shift towards “R600/R607 subunit-containing” AMPAR assemblies. Due to this shift, GluA1(Q600R) and the Q/R-site edited GluA2 subunits are forced to form tetrameric channels composed of subunits that all contain R600/R607. As shown previously, these (R600)-AMPAR tetramers are not processed efficiently and accumulate in the ER (Greger et al., 2002, 2003), and probably access protein degradation pathways faster than Q/R heteromeric channel assemblies as evidenced by the reduced GluA1 level in adult mice.

For both GluA1(Q600R) and GluA2, we visualized the somatic accumulation by immunohistology in brain sections. In *Gria1^R/R^* mice, α-GluA1 as well as α-GluA2 antibodies showed strong immunoreactivity in the str. pyramidale, whereas in *WT* mice immunoreactivity of both antibodies was distributed in the neuropil of the str. oriens and str. radiatum. Nevertheless, the immunogold signal for postsynaptic GluA1(Q600R) and GluA2 showed only a minor reduction of synaptic GluA1(Q600R)-containing and GluA2-containing AMPARs which might represent GluA1(Q600R)/A3 and GluA2/A3 receptors, respectively. However, our immunological analysis cannot exclude a minor sub-population of low conductance (R600/R607)-tetramers in the postsynapse (Lu et al., 2009) as previously suggested (Soto et al., 2007, 2009). The somatic accumulation of GluA1(Q600R) and GluA2 in hippocampi of *Gria1^R/R^* mice was accompanied by a strong reduction of soma currents as analyzed in nucleated patches. Compared to *WT* mice the nucleated patch AMPAR currents were 5–10 fold reduced, indicating that in *Gria1^R/R^* mice the majority of somatic localized GluA1(Q600R)—and GluA2—containing receptors are not delivered to the somatic membrane, or are delivered as homomeric R-containing AMPAR channels with very low single channel conductance (Swanson et al., 1997). In contrast, the synaptic currents of GluA1(Q600R) in CA1 cells were only slightly reduced, in line with the slightly reduced number of GluA1(Q600R) and GluA2 subunits as measured in the immunogold analysis of CA1 synapses.

The impaired distribution of functional AMPARs in CA1 pyramidal cells of *Gria1^R/R^* mice can be monitored by reduced tetanus induced LTP, which is dependent on the pool of extra-synaptic ionotropic glutamate receptors (Granger et al., 2013). As in *Gria1^−/−^* mice, an initial, fast component of synaptic plasticity is missing in *Gria1^R/R^* mice (Zamanillo et al., 1999; Jensen et al., 2003). This strongly suggests that at these synapses this initial potentiation depends on homomeric, Ca^2+^-permeable GluA1-containing AMPARs (Plant et al., 2006; Rozov et al., 2012). In contrast, a longer lasting component of synaptic plasticity that is induced, particularly with the theta-burst pairing protocol, is GluA1 independent (Hoffman et al., 2002; Romberg et al., 2009), and could be induced at CA3-to-CA1 synapses in both *Gria1^R/R^* and *Gria1^−/−^* mice.

The behavioral analysis of *Gria1^R/R^* mice revealed locomotor hyperactivity and a profound spatial short-term memory impairment, but normal spatial reference memory acquisition, both in the Morris water-maze and on the elevated Y-maze, consistent with the learning impairments observed in *Gria1^−/−^* mice (Zamanillo et al., 1999; Reisel et al., 2002; Bannerman et al., 2004) and revealing two distinct forms of spatial information processing (Sanderson et al., 2009, 2010, 2011a,b). First, there is a distinct GluA1-dependent mechanism that supports the efficient performance on working memory tasks such as the spontaneous and rewarded alternation paradigms. Second, there is a GluA1-independent mechanism that allows a long-term association to be formed between a particular spatial location and, for example, an escape platform or a food reward during spatial reference memory tasks such as the Morris water-maze or the Y-maze task, respectively. Although there was some suggestion that the *Gria1^R/R^* mice performed slightly (but not significantly) worse during the acquisition phase of the Morris water-maze task, their performance on the probe test was, if anything, better than that of their *WT* counterparts. This was, in part, due to the fact that as the probe test went on, the *Gria1^R/R^* mice kept searching in the vicinity of the former platform location, whereas the controls started to search elsewhere and thus spent less time searching in the target quadrant. This could reflect differences in the efficiency of the spatial search strategies used by the two groups of mice. Because the *Gria1^R/R^* mice may lack a short-term memory of where they have just recently searched, they may be more inclined to revisit the same spatial location repeatedly during the probe test. In contrast, for the control mice, having visited the former platform location during the initial stages of the transfer test, a sense of relative familiarity for that particular spatial location may bias these mice into searching less familiar areas, elsewhere in the pool in preference to the training quadrant, during the later stages of the probe trial.

## Conclusion

Our results show that the GluA1(Q600R) gene mutation is a loss of function mutation which is capable of evoking the phenotype described for *Gria1^−/−^* mice; a phenotype which is characterized by a specific somatic accumulation of AMPAR subunits, a distinct impairment in synaptic plasticity and selective deficits in spatial working/short-term memory. Thus it seems unlikely that neurological disorders, which exhibit a disturbed AMPAR biogenesis and delivery of synaptic AMPARs (Madeo et al., 2016; Brechet et al., 2017), can simply be explained by somatic accumulation or enrichment of AMPARs, given the highly selective cognitive deficit displayed here by the *Gria1^R/R^* mice.

## Author Contributions

DB designed and analyzed the behavioral experiments. TBorchardt performed the histological and expression analysis of mouse brains and TBus did the statistical analysis for the immunosignal distribution. VJ and NH-Y did the recording and analysis of the field potentials. AR and NB did the nucleated patch clamp recordings and analysis. DZ generated the *Gria1^tm1Erk^* and *Gria1^tm1rsp^* mice. GA and IG were responsible for the quantitative immunogold analysis. RS designed the study and coordinated the experiments. DB, RS, JNR and VJ wrote the manuscript.

## Conflict of Interest Statement

The authors declare that the research was conducted in the absence of any commercial or financial relationships that could be construed as a potential conflict of interest.
