# Supplementary material for: Somatic Accumulation of GluA1-AMPA Receptors Leads to Selective Cognitive Impairments in Mice
Source: Front Mol Neurosci. 2018 Jun 25;11:199. doi: 10.3389/fnmol.2018.00199 (PMC6026654; doi:10.3389/fnmol.2018.00199)
Supplement: Supplementary file 1 [file Data_Sheet_1.DOCX]

Supplementary Figure

Somatic accumulation of GluA1-AMPA receptors leads to selective cognitive impairments in mice

David M. Bannerman^1^*, Thilo Borchardt^2,3^, Vidar Jensen^4^, Andrey Rozov^2,5,6^, Nadia N. Haj-Yasein^4^ , Nail Burnashev^2,7^, Daniel Zamanillo^2,8^, Thorsten Bus^2^, Isabel Grube^9,10^, Giselind Adelmann^9^, J. Nicholas P. Rawlins^1^ and Rolf Sprengel^2,11^*

*** Correspondence:**[David.Bannerman@psy.ox.ac.uk](mailto:David.Bannerman@psy.ox.ac.uk) & [Rolf.Sprengel@mpimf-heidelberg.mpg.de](mailto:Rolf.Sprengel@mpimf-heidelberg.mpg.de)

# Supplementary Figure 1


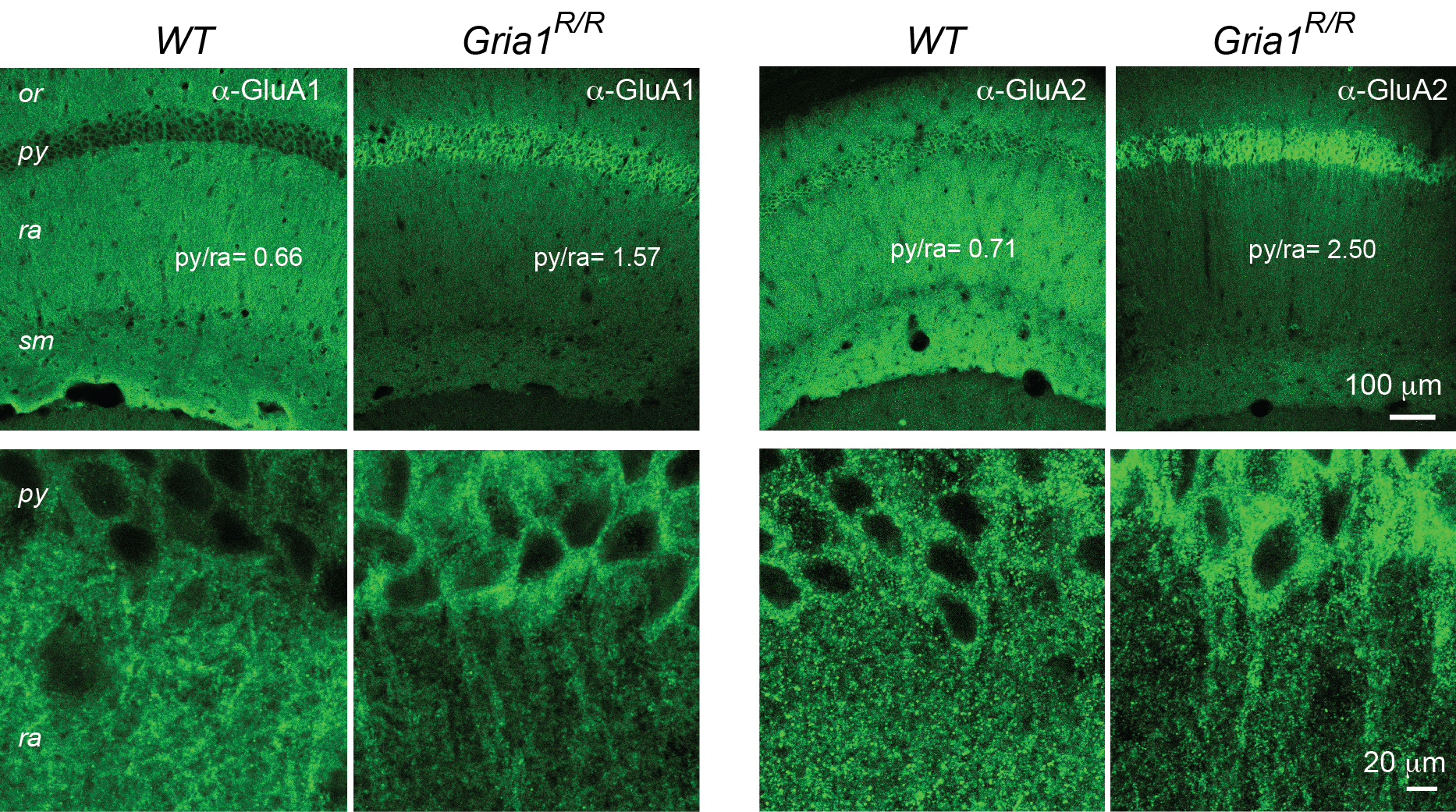


**Supplementary Figure 1.** Confocal images of anti-GluA1 and anti-GluA2 immunstainings of coronal brain sections from wild-type mice (*WT*) and gene-targeted mice (*Gria1^RR^*) that express the GluA1(Q600R) point mutation. The CA1 areas are depicted at different magnifications. The intensities of the GluA1 and GluA2 immunosignals show the somatic GluA1 (Q600R) and GluA2 accumulation in the *str. pyram*idale (py) in hippocampi of *Gria1^RR^* mice compared to GluA1 and GluA2 expression in str. radiatum (ra) and str. lacunumosum moleculare (sm) of *WT* mice. The Somatic Accumulation index (SAi: fluorescence intensity py/ra fluorescence) is indicated.
